# Medical and Philosophical Intelligence

**Published:** 1817-08

**Authors:** 


### MEDICAL AND PHILOSOPHICAL INTELLIGENCE.

*For the London Medical and Physical Journal.*

### *Enquiry on the Re-doubling Pulse;* by Mr. NICHOLAS LITTLETON.

IN the very excellent observations of Dr. Blackall on Hydropic Diseases, it is several times remarked that the patient had a re-doubling pulse. In common language, I believe, to re-double is to repeat or double often. As, 'The hare doubles and re-doubles to avoid the hounds,'—'The wrestler re-doubles his exertions,' *&c.* But the idea intended to be conveyed by the epithet re-doubling, as applied to pulses, I cannot perfectly comprehend; and, as the work alluded to neither contains a description of the term, nor any allusory sentences by which I am enabled, in some measure, to understand its meaning, I should feel obliged to the author, or any other person, for an explanation of that sort of pulse called re-doubling.

It is most likely that the word has been previously used by some other author, who, having given its definition, Dr. Blackall might have thought it unnecessary to repeat it. But, notwithstanding, as it is not a commonly-received term, I hope to see, in a future Number of the Medical and Physical Journal, a description of the redoubling pulse. I wish it more especially, as I have been some time making particular observations on the pulse, perhaps one day to be made public. This question is asked publicly, since it is very unlikely that I should be the only person requiring such information.

*Padstow; June,* 1817.

---

*To the Editors of the London Medical and Physical Journal.*

GENTLEMEN,

IN your last Number, p. 79, you have inserted a communication from the Worcestershire Medical and Surgical Society, relative to an intended application to both Houses of Parliament, praying for a regulation by which the professional man who may be called in to attend a poor unfortunate fellow-creature without any legitimate home, may be entitled to remuneration.

Such a proposition appears to the writer of this letter to be an insult offered to the profession at large. Surely it cannot be intended to infer, that motives so unfavourable to the feelings of humanity can actuate generally the members of a profession which is, or ought to be, of the most humanizing and liberal nature. Those who are in full practice would, no doubt, for the most part, readily and easily extend their aid to a few persons suffering without a home, in poverty and in sickness. Those who are not so fully occupied would surely embrace the earliest opportunity of exerting, with the liveliest interest, those best feelings that animate the human breast, in extending their aid to a poor, sick, and friendless fellow-creature; creature; and, in this last case especially, interest and humanity would go hand-in-hand towards furnishing the relief in question.

Gentlemen,—this is not a theoretical project of humanity: the author of this communication puts in practice, gratis, the principles here recommended, to as many objects, every week, as can require the proposed aid, in the whole county of Worcester, during the same time. Let it not be said that any branch of the profession shall attempt to increase the dreadful amount of the poor-rates, which have already subverted the best principles in the national character of the inhabitants of Britain, and will remain a lasting disgrace to the English nation! Rather let such professional men employ themselves in tilling the earth, and cultivating that soil from which some part of the community may at least be supported, if not benefited.

*London; July* 15, 1817. HUMANITAS.

---

### *Intelligence from America.*

*Prevalent Diseases.*—The hooping-cough has been unusually prevalent and fatal in Boston, during the three last months. In a case of this disease, which was examined after death, the lungs were slightly inflamed on the outside, and uncommonly free from blood within. The mucous membrane of the trachea and bronchiæ was inflamed, and the branches of the bronchiæ full of very white mucus.

The winter epidemic, as it has been called, which has frequently threatened Boston, but never appeared in any great extent, has this season just shown itself and then vanished. A strongly marked case presented itself in the practice of Dr. Jackson. The patient was a boy, ten years old, who was violently attacked with pains in the head and limbs. He had become comatose, and a number of petechiæ appeared, before medical attendance was called for, and he died in forty-eight hours from the invasion. The pulse was, at first, 80; but, within a few hours before death, suddenly changed to 120. On examining the body, the membranes of the brain were found opaque and full of water; the ventricles contained some water. The heart was full of dark fluid blood. The lungs and abdominal viscera were not changed from their healthy state. In another case, occurring at the same time, in a subject of the same age, the boy recovered; but with a paralysis of one side, from which he is not yet well, at the end of two months. During February, numerous cases were observed of sudden attacks of acute pain in a limb, usually shifting to the head and remaining seated there for some time. Pains in the head, especially on one side, and pain in one eye, have been remarkably common. Arising from the same epidemic cause, no doubt, has been the disposition to hydrocephalus, which has been greater than usual. Two cases of this disease may be noticed as presenting something remarkable. The subject of one was a man of about forty, uncommonly strong, active, and industrious. Of late, he had made too free a use of spirituous liquors, and occasionally displayed momentary symptoms of mental alienation, which were attributed to the operation of stimulating drink, especially as he continued to pursue pursue his usual occupations with great ardour, till the moment he was attacked. He was suddenly seized with pain in the breast, cough and expectoration of blood; on the fourth day, he became delirious, and the cough ceased; on the sixth, he was comatose, and died. The body was examined by Dr. Warren, and presented these appearances. The stomach, liver, and other abdominal organs were in a natural state. The heart was large, and full of liquid blood. The lungs dense, inflamed. One or two considerable cysts were found, which had, probably, been formed by a collection of blood from a former occurrence of hæmoptysis; a complaint to which he was occasionally subject. The mucous membrane of the lungs was in a state of high inflammation. On opening the skull, a quantity of water was discharged from the surface of the tunica arachnoidea; this membrane was opaque, and contained a quantity of water between itself and the internal covering of the brain. The surface of the brain was flattened, and exhibited a shrunken or compressed appearance. The veins, on its surface, were small. The substance of the brain was firm. When the ventricles were opened, there appeared, to the surprise of all present, a great quantity of transparent water; and, when the ventricles were emptied, they retained their form perfectly, no doubt, from having been distended for a long time. The quantity of water, collected from the brain, was about eight ounces; but as no preparation had been made to receive it carefully, much was lost. No one will believe, that these appearances of the brain were caused by a disease of five days' duration. It is more probable, that the water had been collecting for a long time, in a very gradual manner. Hence the functions dependent on the brain, were not interrupted, for they had time to accommodate themselves to the change in that organ. This case has been thought more worthy of note, since observing one very similar, lately published by the distinguished Dr. Heberden, in the Lond. Med. Trans. especially, as it is remarked by him, that the brain is not often enough examined, nor its appearances recorded. This case may be considered even more remarkable than that related by Dr. Heberden; since the subject of the latter was a person eighty years old, had been occasionally unwell some time previous to death, though he possessed his intellectual faculties, until the very day on which he died. It is well remarked by Dr. Heberden, 'that since a few drops of blood, a slight puncture or blow on the head, will frequently produce such derangement of the brain as to prove fatal;' we have reason to admire that wonderful power in the animal fabric, of accommodating itself to the greatest changes, when they take place slowly, and by small degrees.

The other case, not less remarkable, is related in the words of the physician who attended.

The child of S. W. aged two years, was attacked on the third day of February, with violent symptoms of fever. After a few days, the febrile symptoms subsided, leaving the child in a state of stupor, which was now and then interrupted with violent shrieks, and appearance of much distress and agitation. The head was constantly rolling from side to side; the occiput inclined backward. The eyes were dim, pupil insensible to light, and somewhat dilated. Vision was evidently impaired or lost, as no notice was taken of objects approximated to the eye. The sense of hearing was thought to be diminished, and might be wholly lost. The left arm and leg remained immoveable; the opposite extremities were occasionally agitated. The tongue, at first, foul, afterwards became clean. The intestinal evacuations were always of a green mucus; but never occurred except from the use of purgatives; drinks were swallowed, when presented, in a wild, idiotic manner.

These symptoms resisted the use of the most powerful remedies; and about the 14th day appeared to indicate a confirmed hydrocephalus, of the most distinct character. All medicines were, therefore, omitted, and the parents resigned themselves to the loss of their child, which was daily expected. In fourteen days after, I was called to the house, and, to my great surprise, found the child still living, though insensible to external objects, yet seeming to suffer pain, as it occasionally shrieked in the most distressing manner. The pulse was slow, intermittent. The skin cold and loose from great emaciation. The mother was advised to give opiates, in order to keep the child easy, and to relieve its bowels periodically by purgatives. On a second visit, I found the pulse regular, though very slow. The eyes now and then open, and presenting a strange appearance from the enlarged state of the pupil. The hopes of the mother were revived, and I felt sufficiently encouraged to recommend to blister the head anew, and to give a purgative dose of calomel, with jalap, every other day. On the operation of these medicines, a rapid amendment took place. This child, after remaining insensible to external objects for nearly four weeks, began to notice things around it; soon after to reach out to the spoon: then it attended to things calculated to amuse it, began to smile and stretch out the hand. It appears perfectly sensible now. Eats freely of solid food, and gains strength daily. The pulse continues very slow. The skin is cold. The pupil is very much dilated; but contracts, on turning the head to the light.

For a few days, there has been a slight discharge of matter from the left ear; but I cannot discover any other mark of disease in the external auditory passage.

There has not, at any time, been the slightest appearance of worms, living or dead; nor of the remains of worms. The discharges have been uniformly of a green mucus, as well during, as after the operation of the most powerful purgative, until since the other changes in the symptoms of disease. They are now quite healthy, yet appear only in consequence of taking purgatives.

Since the above was written, the intestines have begun to perform their healthy functions without medicine. The child uses its legs, can stand a little, and appears well, except that it is still feeble. The pupil of the eye remains dilated.

There cannot, in my opinion, be a question as to the propriety of considering considering this as an instance of recovery, from a collection of water in the brain.

*Lithotomy.*—An accident occurred to Dr. Warren, in performing the operation of lithotomy, which it may be useful to record. The patient was about fifty years of age; of a very gross habit, and relaxed fibre; had laboured under his complaint for many years, during the last two or three of which, he had been endeavouring to prepare his mind for the operation. His sufferings became, at length, so excruciating and so constant, that he consented to take the risk of having the stone extracted, although the state of his system, and the size of the stone, contributed to lessen the probability of success. On sounding him, the stone was readily perceived; but could not be directly struck by the instrument, which appeared rather to rub it in passing, than to strike it. On examining by the rectum, both in the upright and horizontal posture, the stone could not be perceived. The patient being properly prepared, the external incision made, and the urethra opened, the gorget was passed along the staff; but, before entering the bladder, was found to strike hard upon the stone, and its further passage was impeded. The direction of the staff was then altered; and the gorget, being pushed steadily on, and carefully kept to the staff, overcame the resistance, and a stream of urine immediately appeared. The gorget being felt to strike the stone, the staff was withdrawn; but, on passing the finger along the gorget, it appeared that the opening into the bladder was too small to be perceived in so deep a wound. The gorget was withdrawn, and a round sound was passed through the urethra into the bladder; then a female staff was passed through the wound till it struck the sound, where the urethra was opened, and, by means of the sound, was conducted into the bladder, and the stone was struck with it. Then a curved knife was passed along the groove of this staff into the bladder, and both, being turned to the left, a free incision was made in the bladder. The forceps was then guided by the staff to the bladder; the wound being too deep to admit of the finger's reaching the bladder. The shortest diameter of the stone was found, and then it was drawn to the wound, but was found too large to pass through it. A bistoury was, therefore, passed to the stone, and insinuated between it and the bladder, so as to cut the external angle of the wound; and then, by gradual movements, the stone was brought out, without the use of great force. On examining the stone, it was found to be large, smooth, except at one part, where for about an inch, it was rough and covered with transparent crystals, to some of which were attached small portions of membrane. At the largest end of the stone, was seen the mark made by the beak of the gorget; and, on examining the instrument, it appeared, that about a quarter of an inch of its edge was knocked off, where it had struck the stone. The patient did well on the three days following the operation. On the fourth, the abdomen became tense, and occasionally painful. His strength diminished rapidly, and he sunk on the seventh day. The body was examined by Dr. Warren, in company with Dr. Jackson. The swelling of the abdomen was found to arise wholly from air contained tained in the large intestines. A slight blush from inflammation was diffused through these intestines. The peritoneum, over the upper part of the bladder, was discoloured. The bladder had, internally, a dark coloured appearance, and, at the part corresponding with the symphysis pubis, the mark of adhesion of the stone was observed. Extending from this part to the left side of the pelvis, appeared some purulent matter, in the copious cellular membrane, which enveloped the bladder. The incision of the bladder was found to be as exact as possible, through the left half of the prostate gland, and three-quarters of an inch of the bladder. This organ was not wounded, torn, nor hurt in any other part. The rectum, on its fore-part, was in perfect state. On the part attached to the coccyx, it was discoloured from a small portion of coagulated lymph. The external wound, and the scrotum, were dark coloured and gangrenous. The stone, being measured, was found to be nearly three inches in length, and five in diameter.

There are many histories of stones being inclosed in a sac of the bladder; but there was nothing of the kind here. The impossibility of discovering the stone by the rectum, the resistance opposed to the gorget, the appearance of the stone, and the appearance of the inner coat of the bladder, all concur in showing, that this stone adhered strongly to the fore-part of the bladder; from which it was separated by the stroke of the gorget. The separation of the stone from the bladder was the cause of the inflammation of the peritoneum covering it; and this inflammation, propagated by sympathy to the colon, was the cause of death; although the inflammation was not so considerable but that the patient would have recovered, if his constitution had not been impaired by disease and confinement. The gangrenous appearance of the scrotum and external wound was not produced by contusion, as there was none applied to those parts, and the pressure, on the internal wound, was not so great as that often employed in cases which terminate well.

The peculiar occurrence, in this case, forms an objection to the use of the gorget. The operator was led to it, in this instance, from observing, that in three preceding cases, in which he had used the knife, the cures were more slow than where the gorget had been used; and also, from a comparison of the wound, made by the gorget and by the knife, in dead bodies, operated on and dissected for the purpose of this comparison. The wound, made by the knife, was never so exact, as to size nor situation, as that made by the gorget. Nor can it be, since the gorget is always conducted by the staff, while the knife necessarily quits the staff to make the incision of the prostate gland.—*New-England Journal.*

---

*(From Paris.)*

***Observations on a Strangulation of the Small Intestines, terminated by Gangrene and Death;*** by M. Bougon, Surgeon to Monsieur.—The records of medicine present no example of an accident similar to the present; but this may be only for want of that attention to morbid anatomy, in which moderns have so many advantages. On the 13th of Dec. 1816, Augustus-Hyppolite Mallet, groom, aged 19 years, having always had a good state of health, obliged all day long to ride behind a cabriolet, felt a slight colic, which augmented towards evening: the pain increased a little during the night and on the morning of the 14th; and, at half-past four o'clock, P. M. M. Regnault, Consulting-Physician to the King, was consulted. The symptoms then were, colic not very violent; abdomen tender to the touch; pulse much accelerated, full, and regular; acute fever; face and eyes animated. Emollient fomentations on the belly, an infusion of linden-flowers for drink, glysters, with the decoction of linseed and oil, were prescribed, but without affording any relief; and, shortly after, vomitings took place. On the 15th, at eleven o'clock in the morning, having visited the patient in the absence of M. Regnault, we found the face Hypocratic, much swollen; the pulse concentrated, irregular, intermittent; the belly swollen, very painful at the navel; the extremities cold and livid: in short, some moments afterwards the patient expired, vomiting abundantly a very deep-coloured greenish serum, of a most fetid odour; and this discharge continued some time after death.

"On the 16th, at ten o'clock in the morning, we proceeded, with M. Le Blanc, Physician of the 1st Arondissement, and in the presence of Dr. Regnault, to the opening of the body, which was little altered. 1. Abdominal cavity, which was swollen by very fetid gases. 2. The effusion of several pints of blackish serum, very fetid, in the peritoneal cavity. 3. Many gangrenous points in different parts of the peritoneum, and principally at the gastro-colical epiploon. 4. All the small intestines inflamed, and partly gangrened. 5. This last kind of alteration still more striking at the end of the ilium intestinum, and at the kind of digital appendage comprehended in the extraordinary strangulation, formed by the piece, which we have detached with care, and which we have thought it our duty to submit to the examination of the faculty. The parts most altered were seated on the right side of the hypogastrium, before and behind the psoas. 6. The cœcum, the cœcal appendage, the colon, and the rectum, participated but little in the inflammation of the other parts of the intestinal tube; and the internal membrane of the stomach was completely exempt from it."

*Description of the Anatomico-Pathological Piece, relative to the preceding Observations;* by Messrs. A. Beclard and Jules Cloquet.—We had at first great difficulty to recognise the disposition of the parts interested in this strangulation, from the degree of constriction of the knot which formed it, and the swelling of the intestines. However, we succeeded in undoing this knot, and we found that the *ilium intestinum* gave birth to a pediculated appendage, six inches long, very flexible, and which occasioned the strangulation by forming a knot around a convolution of the same intestine. At the place where the appendage detached itself from the intestine, the cavity of the latter was a little narrowed. The convolution of the intestine strangulated by this appendage was a foot and a half long. This appendage, having passed behind the intestine, and afterwards bent backwards on itself, had crossed, in passing under and near the commencement of the intestine. In this manner it had formed a real knot, which embraced the intestinal convolution and its mesentery. Of two ends of the intestine, situated above the knot of the appendage, the upper one was red, inflamed, and much dilated; the lower one was pale, shrivelled, and offered no trace of inflammation. The strangulated intestine was red, much dilated, and filled with about a pint of black and fetid blood, very fluid. The appendage was full of a similar liquor, but which could not flow into the intestine, even by a very strong pressure, because the knot stopped the passage. The membranes of the intestine were red, and as if injected with blood. The knot of the appendage must have been recent, for it had made no impression on the intestine, which resumed its usual size, from the moment when we had untied this artificial ligature.

---

*Royal Society of Edinburgh.*—On the 19th of May, a paper by Mr. STEVENSON, civil engineer, was read, regarding the operation of the waters of the ocean and of the river Dee, in the basin or harbour of Aberdeen; from which it appears that Mr. Stephenson, in the month of April, 1812, with the use of an instrument (of which he exhibited a drawing), has been able to lift salt water from the bottom, while it was quite fresh at the surface, and has satisfactorily ascertained that the tidal or salt waters keep in a distinct stratum or layer under the fresh water of the river Dee. This anomaly, with regard to the salt and fresh waters, appears in a very striking manner at Aberdeen, where the fall of the Dee is such as to cause river water to run down with a velocity which seems to increase as the tide rises in the harbour and smooths the bed of the river. These observations show that the salt water insinuates itself under the fresh water, and that the river is lifted *bodily upwards*; thus producing the regular effect of flood and ebb tide in the basin, while the river flows downward all the while with a current which for a time seems to increase as the tide rises.

These facts, with regard to the continual course of the river Dee downward, is such a contrast to the operation of the waters of the Thames, as seen by a spectator from London Bridge, that Mr. Stevenson was induced to extend his experiments to that river in the years 1815 and 1816, by a train of experiments and observations from about opposite to Billingsgate all the way to Gravesend.

The waters of the Thames, opposite the London Docks' gates, were found to be perfectly fresh throughout; at Blackwall, even in spring tides, the water was found to be only slightly saline; at Woolwich the proportion of salt water increases, and so on to Gravesend. But the strata of salt and fresh water is less distinctly marked in the Thames than in any of those rivers on which he has hitherto had an opportunity of making his observations. But these inquiries are meant to be extended to most of the principal rivers in the kingdom, when an account of the whole will be given.

 From

From the series of observations made at and below London Bridge, compared with the river as far up as Kew and Oxford, Mr. Stevenson is of opinion that the waters of the Thames seldom change, but are probably carried up and down with the turn of the alternate tides for an indefinite period, which he is of opinion may be one, if not the principal, cause of what is termed the extreme softness of the waters of the Thames.

Mr. Stevenson has made similar experiments on the rivers Forth and Tay, and at Loch Eil, where the Caledonian canal joins the western sea. The aperture at Curran Ferry for the tidal waters of that loch being small compared to the surface of Loch Eil, which forms the drainage of a great extent of country. It, therefore, occurred to Mr. Stevenson that the waters of the surface must have less of the saline particles than the waters of the bottom. He accordingly lifted water from the surface at the anchorage off Fort William, and found it to be 1008·2; at the depth of nine fathoms, 1025·5; at the depth of 30 fathoms, in the central parts of the loch, it was 1027·2; indicating the greater specific gravity, and consequently more of the saline parts, as the depth of the water is increased.

---

*Royal Geological Society of Cornwall.*—At the quarterly meeting of this society a paper was read, on the different Tests for the Discovery of the Presence of Arsenic, by Dr. PARIS. The author stated, that, since the extraordinary and notorious trial at the late assizes, his opinion had been so repeatedly solicited upon the subject of arsenical tests, that he felt it his duty to offer the present paper as an answer to them. It afforded him also an opportunity of communicating to the society a simple method of so modifying the ordinary experiments as entirely to avoid those fallacies which had been attributed to them. The test of nitrate of silver was well known to furnish its indication by the colour of the precipitate which it induced with the suspected liquid. It had, however, been observed by a pupil of Dr. Marcet that the phosphoric salts had the property of throwing down with nitrate of silver a precipitate perfectly analogous in colour to that from arsenic; and, as these salts were known to have existence in the animal fluids, a source of perplexity and error was thus connected with any experiment, with nitrate of silver, on the contents of the stomach. This difficulty, however, the author stated might be overcome by modifying the experiment as follows:—Instead of conducting the trial in glasses, drop the suspected liquor upon writing paper, making a broad line with it. Along this line a stick of lunar caustic is to be slowly drawn, when a streak is produced of a colour resembling that known by the name of the *Indian yellow*, and this alike obtained by the presence of arsenic and of phosphoric salts; but the one from arsenic is rough and curdy, as if effected by a crayon; the other, quite smooth and even in its appearance, such as would be produced by a water-colour. A more important, and still more unequivocal, mark of distinction soon succeeds; in less than two minutes the phosphoric yellow fades into a

"*sad*

"*sad green*," becoming gradually darker until it becomes black; the arsenical yellow, on the other hand, remains permanent for some time, when it becomes brown. In performing these experiments, the sun-shine should be avoided, or the transition of the colour is too rapid. This experiment, however, is not related with a view to supersede the more important one of the reduction of the metal; indeed, in a matter of such serious importance, observed the author, a combination of unequivocal proofs was required. Mr. Gregor had suggested to him the application of a nitrate of titanium as a new test. In this case the suspected powder should be treated with nitric acid. The circumstance of the phosphoric acid precipitating the titanium in a manner similar to arsenic, offered an objection which he was not prepared to surmount. It was, however, well worthy the attention of chemists.

---

*Further Improvements in Professor Leslie's Method of producing Ice.*—Extracted from the Annals of Philosophy.

*(To Dr. Thomson.)*

DEAR SIR, *Edinburgh, May* 20, 1817.

I think it worth while to mention, in this stage of my experiments, that parched oatmeal has a stronger and more extensive power of absorbing humidity than even the decayed trap rock. With about three-quarters of a pound of meal, occupying a surface of seven inches in diameter, I froze nearly a quarter of a pound of water, and kept it for the space of twenty hours in the form of ice till one-half of the congealed mass was again melted. The temperature of the room being nearly 50°, the meal had then absorbed the eighteenth part of its weight, though it had not yet lost more than one-third of its desiccating power. With a body of dried oatmeal a foot in diameter, and rather more than one inch deep, I have since frozen a pound and a quarter of water contained in a hemispherical porous cup, and, though the room is warmer than before, the energy of absorption seems to be capable of maintaining the state of congelation for a considerable time. It is curious to observe that when the experiment was reversed, and the surface of the water about double that of the meal, this substance acquired, after the air under the receiver had been rarified, a heat exceeding 50° of Fahrenheit, so as to feel, indeed, sensibly hot on applying the hand.

I am, dear Sir, sincerely yours,

JOHN LESLIE.

---

*Singular Formation found within an Egg.*—"This formation was discovered floating in the white part of a common egg. The outside consists of a shell exactly similar to that of the egg itself, but not so thick, and of a darker colour. It is about one inch and three quarters in length. Its greatest diameter, which is near the upper end, is about three quarters of an inch, and it tapers down to a point at the other end of an eighth of an inch in diameter. By piercing it at the top, and at the bottom, I was enabled, by applying my mouth at one end, to force out the contents, which I found found exactly similar to the white of an egg, but there was no yolk. The shell gradually becomes thinner as it approaches the other end. In the centre of it, on the surface, there is an indentation, or ring, which extends a little more than half round the egg; and about a quarter of an inch from the narrower end there is another indentation, which extends almost the whole of the way round. The egg in which this singular curiosity was found was good in every other respect, and had in it a perfect yolk."—*Annals of Philosophy.*

---

The Report of the *National Vaccine Establishment* gives the usual favourable account of its progress; and, in answer to some unfavourable reports in the county of Essex, concludes thus:—

"In consequence of the reported failure of vaccination in the parish of St. Osyth, we, the under-mentioned medical gentlemen, on the recommendation of the Rev. Archdeacon Jefferson, and the overseers of the same parish, have fully and deliberately, this day, investigated the matter; and beg, for the satisfaction of the public at large, to make the following declaration.

"We consider that the small-pox has, in very few instances, supervened to vaccination; but that, in those instances, we are of opinion that vaccination, most probably, had not been perfectly introduced into the system, owing simply to the nature and progress of the disease not having been at that time thoroughly understood.

"In the remainder of the cases we have witnessed, we are of opinion, that, although some cutaneous eruptions had taken place, they were by no means decidedly variolous, and, if any of them did put on that appearance, they were of a mild and transient nature.

We therefore wish to declare, that we shall feel anxious to continue to prosecute vaccination, considering it one of the most valuable discoveries to society.

"James Moore, Esq. Director of the National Vaccine Establishment, accompanied us during this investigation, and is precisely of the same opinion with us, and is to report the particulars to the Institution upon his return to London.

*St. Osyth;*

*May* 14, 1817.

GEORGE ROGERS.

ROGER NUNN.

MAURICE MASON.

THOMAS OSMOND.

ROBERT MARTIN."

We have received an equally favourable account of the progress in France. A case of variola post vaccinationem is admitted, and the proper remark follows, that similar instances have occurred after small-pox.

---

A General Meeting of the Subscribers to the Association of Apothecaries and Surgeon-Apothecaries of England and Wales is announced for the 20th of this month.—See our Wrapper.

A METEO-

### A METEOROLOGICAL JOURNAL,

### By Messrs. William Harris and Co. 50, Holborn, London.

*From the 20th June to the 19th July, inclusive.*

| Day of Month. | Rain gauge | Therm. | | | Barom. | | De Luc's Hygrom. Dry | | De Luc's Hygrom. Damp | | Wind. | | Atmospheric Variation. | | |
|---|---|---|---|---|---|---|---|---|---|---|---|---|---|---|---|
| June | | | | | | | | | | | | | | | |
| 20 | | 72 | 82 | 69 | $29^8$ | $29^7$ | 0 | 5 | 0 | | SE | E | Fine | | |
| 21 | | 74 | 83 | 63 | $29^8$ | $29^9$ | 0 | | 0 | 2 | SE | E | Fine | | |
| 22 | | 69 | 82 | 61 | 30 | 30 | | | 3 | 2 | N | N | Fine | | |
| 23 | | 68 | 80 | 63 | $29^9$ | $29^9$ | | 2 | 1 | | N | N | Fine | | |
| 24 | | 67 | 79 | 60 | $29^9$ | $29^9$ | 3 | 0 | | 0 | WSW | WSW | Fine | | Showery |
| 25 | | 63 | 75 | 61 | 30 | 30 | 0 | 0 | 0 | 0 | WSW | SW | Fine | | |
| 26 | | 66 | 79 | 64 | 30 | $29^9$ | 0 | 0 | 0 | 0 | SW | S | Fine | | |
| 27 | | 64 | 75 | 63 | $29^6$ | $29^6$ | | | 1 | 1 | NE | SW | Fine | Rain | Fine |
| 28 | | 61 | 71 | 60 | $29^6$ | $29^5$ | 2 | 2 | | | SW | SW | Fine | Rain | Clo. |
| 29 | | 63 | 70 | 61 | $29^5$ | $29^7$ | 3 | 2 | | | WSW | SE | Fine | | Rain |
| 30 | | 64 | 69 | 59 | $29^6$ | $29^3$ | 2 | 0 | | 0 | S | S | Clo. | Fine | Clo. |
| July | | | | | | | | | | | | | | | |
| 1 | | 62 | 68 | 57 | $29^3$ | $29^4$ | | | 1 | 2 | SE | SW | Rain | | Clo. |
| 2 | .09 | 61 | 67 | 53 | $29^7$ | $29^9$ | | | 1 | 1 | SW | SW | Clo. | Fine | |
| 3 | .10 | 58 | 67 | 55 | $29^7$ | $29^6$ | | | 1 | 2 | S | S | Fine | Rain | |
| 4 | .11 | 57 | 63 | 59 | $29^6$ | $29^5$ | | | 3 | 5 | SE | SW | Rain | Clo. | Fine |
| 5 | .10 | 61 | 67 | 53 | $29^5$ | $29^7$ | | | 5 | 5 | SW | SW | Clo. | Rain | Clo. |
| 6 | .07 | 56 | 64 | 53 | $29^7$ | $29^7$ | | | 5 | 5 | SW | SW | Rain | Clo. | Fine |
| 7 | 03 | 60 | 69 | 54 | $29^7$ | $29^8$ | | | 3 | 3 | SW | SW | Fine | Rain | Fine |
| 8 | .04 | 59 | 70 | 54 | $29^8$ | $29^8$ | | | 3 | 3 | W | W | Fine | | |
| 9 | | 59 | 70 | 55 | $29^8$ | $29^7$ | | | 2 | 2 | SW | SW | Fine | | |
| 10 | | 57 | 68 | 53 | $29^7$ | $29^7$ | | | 2 | 2 | SW | W | Fine | | |
| 11 | | 56 | 68 | 51 | $29^7$ | $29^6$ | | | 2 | 3 | SW | SW | Clo. | Fine | |
| 12 | | 59 | 67 | 48 | $29^7$ | $29^7$ | | | 3 | 3 | W | SW | Fine | Clo. | Fine |
| 13 | | 58 | 68 | 47 | $29^8$ | $29^8$ | | | 5 | 5 | W | SW | Fine | Clo. | Fine |
| 14 | | 59 | 66 | 49 | $29^8$ | $29^7$ | | | 5 | 4 | SW | SW | Clo. | | |
| 15 | .06 | 60 | 66 | 52 | $29^7$ | $29^7$ | | | 3 | 4 | SSW | SW | Rain | | Clo. |
| 16 | | 58 | 67 | 51 | $29^6$ | $29^5$ | | | 3 | 3 | W | W | Clo. | Rain | Clo. |
| 17 | ,05 | 63 | 69 | 54 | $29^6$ | $29^6$ | | | 3 | 3 | W | W | Fine | | |
| 18 | | 62 | 69 | 57 | $29^4$ | $29^8$ | | | 3 | 3 | W | W | Fine | | |
| 19 | | 63 | 71 | 56 | $29^9$ | $29^9$ | | | 3 | 3 | W | W | Fine | | |

The quantity of rain fallen in the month of June cannot be correctly ascertained, the gauge having leaked the latter part of that month, but it is supposed to be about 1 inch and 60-100ths.

### REPORT OF DISEASES.

THE season continues healthy, and many chronic diseases have been much alleviated, some entirely relieved, after having been first superseded by complaints in a more acute form.

Patients under phthisis *burn out* rapidly in this genial atmosphere, but are more free from general uneasiness, and more sanguine in their prospects of recovery. This is no inconsiderable relief to the practitioner who has these most interesting subjects perpetually before him.

The few fevers which have appeared have been more or less inflammatory, and with difficulty reduced to laws, or are, to use the French term, *ataxique*. They have rarely proved fatal, but extremely tedious if the early symptoms were not relieved by bleeding. It has not, however, been necessary to carry this practice to any great extent, but, if omitted in the beginning, it was necessary to repeat it in almost every stage of the subsequent progress; convalescence proved extremely slow, and in all these cases the patients became œdematous.

